# Eighteen-year follow-up report of the surveillance and prevention of an HIV/AIDS outbreak amongst plasma donors in Hebei Province, China

**DOI:** 10.1186/s12879-015-1073-y

**Published:** 2015-08-06

**Authors:** Suliang Chen, Hongru Zhao, Cuiying Zhao, Yuqi Zhang, Baojun Li, Guangyi Bai, Liang Liang, Xinli Lu

**Affiliations:** Hebei Province Center for Disease Control and Prevention, 97 Huaian East Road, Yuhua District, Shijiazhuang, 050021 China

**Keywords:** HIV, Plasma donor-related HIV infection, Couple transmission, Mother-to-infant transmission, Dynamic tracing, Antiviral therapy

## Abstract

**Background:**

There has been a clear increase in HIV-1 infection cases in recent years in Hebei Province, China, and transmission via blood is one of the risk factors in the early. This article aimed to investigate the HIV infection rate and control efficiency among the paid blood donor population over a period of 18 years.

**Methods:**

From 1995–2013, HIV/AIDS cases among former blood donors in Hebei Province were registered and closely monitored to collect data of all-cause mortality, intervention measures to prevent family transmission, disease transmission between couples as well as between mothers and infants, and HAART therapy outcomes.

**Results:**

A total of 326 cases were identified as directly infected with HIV/AIDS during plasma donation in Hebei Province. Of these, 146 cases (44.8 %) were identified in the same year as infection; 180 cases (55.2 %) were identified 1–18 years after infection because they did not participate in the 1995 screening. The final case was identified in February 2012. By 2013, the mortality rate and survival rate of plasma donor-related HIV/AIDS was 54.9 % and 45.1 %, respectively. The identified transmission rate between couples was 11.3 % (8/71); this rate during the same year as infection was 3.3 % (1/30), and the rate 4–17 years after HIV infection was 17.1 % (7/41). Approximately 91.2 % (145/159) of married women of childbearing age did not have children after being informed of HIV infection. Only 8.8 % (14/159) of these women had children after being informed of HIV infection. The mother-to-infant transmission rate was 38.5 % (5/13).

The HAART coverage rate has increased from 10.1 % (16/159) in 2003 to 83.6 % (127/152) in 2013. Since 1999, the HIV mortality rate has trended up; by 2013, the cumulative mortality rate reached 54.9 % (179/326). After HAART was initiated in China, the death rate decreased to some extent. Second generation transmission (via couple or mother-to-infant transmission) among blood donor-related HIV cases accounted for approximately 4.0 % (13/326). All first- or second-generation cases were infected with HIV-1 subtype B.

**Conclusions:**

In this accident of HIV-infection among plasma donors in Hebei Province, a total of 339 direct and second-generation cases have been identified over 18 years of monitoring. Favorable clinical results have been achieved using intervention measurements and antiviral therapy.

## Background

In the early 1950s, there were many blood collection stations throughout the city of Langfang in Hebei Province, China. These stations were regarded as the blood donation base to replenish blood supplies for Beijing and Tianjin. The 1980s saw the introduction of new technology to China that enabled single-donor plasma collection. Following this, plasmapheresis centers were established in this region and gradually spread to other regions within Hebei Province, predominantly in towns and villages where a large number of paid donors could be sourced. The recruited medical workers generally had little formal training or background in blood collection. Tragically, cross-infection frequently occurred during various steps of plasma isolation, such as during initial blood collection, plasma separation, and blood cell return to the donor. The prevalence of hepatitis C, malaria, and HIV/AIDS among paid donors at these plasmapheresis centers increased beginning in 1985 [1, 2], 1993 [3], and 1995 [4], respectively. Given the prevalence of cross-infections, particularly of HIV/AIDS, all plasmapheresis centers across the entire province were closed. We have monitored HIV/AIDS cases and deaths up to 2013, investigating rates of family transmission, preventive interventions, and HAART during the past 18 years. This study was conducted to acquire data on the prevalence of HIV/AIDS among cross-infected plasma donors in Hebei Province.

## Methods

### Surveillance

#### Collective screening

After early cases of HIV-infected plasma donors were discovered in 1995 in Langfang, physical examinations were performed and HIV antibody levels measured for all plasma donors throughout Hebei Province. From 26 November 2004 to 15 February 2005, in compliance with notification from the General Office of the Ministry of Health, registration and blood sampling of former paid blood donors was repeated, so that HIV antibody screening could be carried out.

#### Daily surveillance

Through enactment of policies offering freely accessible voluntary counseling and testing (VCT), expanded testing within medical institutions, and monitoring the spouses and children of HIV-positive individuals, infected donors from the population who did not participate in the initial HIV antibody screening were identified. These individuals had not been screened because they had failed to participate from fear of discrimination; instead, they were screened during treatment for other diseases. To encourage former paid donors to undergo testing, in January 2012, the government enacted a living subsidy policy for people who acquired HIV via transmission through blood.

#### Source surveillance

Plasma samples from blood plasma donors between 1990 and 1994 were acquired from serum banks and screened. The samples included 140 cases collected from 1990 to 1991, 1090 cases from 1992 to 1994, 124 cases from 1993 to 1994, and 699 cases in June 1994. The transfusion histories of four HIV cases identified in 1995 were investigated, and recipients of blood from plasma donors in Hebei Province were retrospectively followed.

#### Death surveillance

For the identified HIV/AIDS cases, half-yearly follow-up examinations were conducted. Registered HIV/AIDS deaths were determined from all-cause mortality (i.e., including AIDS and non-AIDS related deaths). A portion of the paid blood donors from Hebei Province were not investigated or followed up because they no longer resided in the province. Additionally, some donors were not tested for HIV, even though they continued to reside in Hebei. Therefore, it is possible that the number of HIV infections could be underestimated.

### Cases

Blood samples were collected from identified HIV cases. Blood serum was separated for all samples, for preliminary screening using enzyme-linked immunosorbent assay (ELISA). All positive HIV antibody test results were confirmed by western blot assay. HIV-positive cases with a history as former plasma donors and that were confirmed by blood tests, excluding other transmission risk factors, were determined to have been infected via cross-infection during donation of blood at plasmapheresis centers in Hebei Province. If these individuals had one or more AIDS-indicative diseases or a CD4 count <200 cells/mm^3^, they were diagnosed as plasma donor-related AIDS cases. For identified cases of HIV/AIDS, spouses who tested positive for HIV antibody, excluding risk factors of plasma donation, extramarital sexual behavior, intravenous drug use, blood transfusion and so on, were categorized as couple transmission. Female donor-related HIV/AIDS cases who were pregnant, who had normal vaginal delivery, and/or who breastfed after infection, and whose infants tested positive for HIV antibody 18 months after birth, excluding other transmission factors, were categorized as mother-to-infant transmission.

### Prophylaxis and treatment

Spouses of all identified plasma donor-related HIV/AIDS cases were subject to sample collection and testing if they were married. In cases negative for HIV antibody, intervention and prevention measures were carried out, such as provision of condoms as well as dynamic tracing. All children of female cases had the possibility of mother-to-infant transmission, and were subject to sample collection for further testing. During regular follow-up of identified HIV-positive individuals, all cases meeting the diagnostic criteria for AIDS, or with CD4 counts <200 cells/mm^3^, were treated with highly active antiretroviral therapy (HAART).

Beginning in 2008, cases with CD4 counts ≤350 cells/mm^3^ were also treated with HAART. During the period 1995–2002, HAART was unavailable in China so AIDS patients did not have the opportunity to receive this form of therapy. From 2003, the Chinese government began a policy of providing free HAART, which included a non-nucleoside reverse transcriptase inhibitor (EFV) and two types of nucleoside/nucleotide reverse-transcriptase inhibitor (AZT, 3TC, d4t, ddl) in combination therapy. In 2007, second-line drugs (LPV/r and TDF) were introduced, which enabled timely replacement of first-line or other second-line regimens in drug-resistant patients or for those suffering toxic side effects.

### Genotype detection

In seven plasma donor-related HIV cases (designated as heb0202, heb0203, heb0206, heb0208, heb0209, heb02012, heb02015), nested PCR was used to amplify the HIV-1 *env* gene. The amplified products were sequenced and used to create a phylogenetic tree using the neighbor-joining method.

### Statistics

A database of plasma donor-related HIV/AIDS cases and their family members was created. Prior to statistical analysis, the detection data and original records in the database were checked thoroughly. Follow-up analysis was performed, if necessary, such that all cases that did not meet the conditions were excluded; those that met the conditions were transferred to the final database. SPSS 15.0 and Excel were used to perform the statistical analysis. Analyzed data included that of the HIV outbreak and source, annual changes in HIV/AIDS cases, sexual transmission between couples, and mother-to-infant transmission and so on.

### Ethics statement

All investigations and HIV-1 tests were informed and voluntary, and participants agreed that their samples may be used for the purpose of controlling and preventing HIV. Written informed consent was obtained from all adult patients and parents/guardians of HIV-1 positive children in this study. The study was conducted according to the Declaration of Helsinki and was approved by the local Ethics Committee at Hebei Province Centers for Disease Control and Prevention, ethics board document number IRB(P)2014-002.

## Results

### HIV outbreak and source

In February 1995, a paid plasma donor in Langfang, Hebei Province was identified as HIV-1 positive during screening for blood donation at a hospital in Tianjin. From February to April of 1995, HIV screening of former paid plasma donors was conducted throughout Hebei, and the results indicated that the HIV infection rate was 1.5 % (146/9811) among paid blood donors. In the same year, all plasmapheresis centers in Langfang and across Hebei Province were closed. Donors’ blood donation histories were retained during the HIV screening process, owing to concerns from some donors that they would be discriminated against if identified as HIV-positive. Some donors who did not participate in the screening eventually due to worry about discrimination. With improvements in the HIV monitoring network from 1996 to 2013, additional HIV-positive plasma donors were identified. Source monitoring indicated that 2053 former paid blood donors were identified as being HIV-negative between 1990 and 1994. Four individuals who tested positive for HIV in 1995 were found to have been infected with HIV through blood transfusions (no other risk factors) received in October 1994; the blood donor was HIV-negative at the time of supplying the blood, but later tested HIV-positive [5]. Blood specimens from 45 blood recipients who received blood transfusion from January 1993 to September 1994 tested as HIV-negative.

Regarding the causes of the HIV outbreak in Hebei Province, before 1995, HIV-positive individuals could enter the blood plasma collection process with no required HIV testing. Single-donor plasma collection included two cycles of whole blood collection, plasma separation and blood cell return to the donor. Physiological saline, medical scissors, hemostatic forceps, centrifuges, catheters and other instruments were shared among plasma donors. Especially in the plasma separation room, the sharing of instruments between donors and medical workers results in widespread cross-contamination of blood plasma in the plastic catheters connected to blood bags used during the process of plasma separation.

### General information

**Fig. 1:**
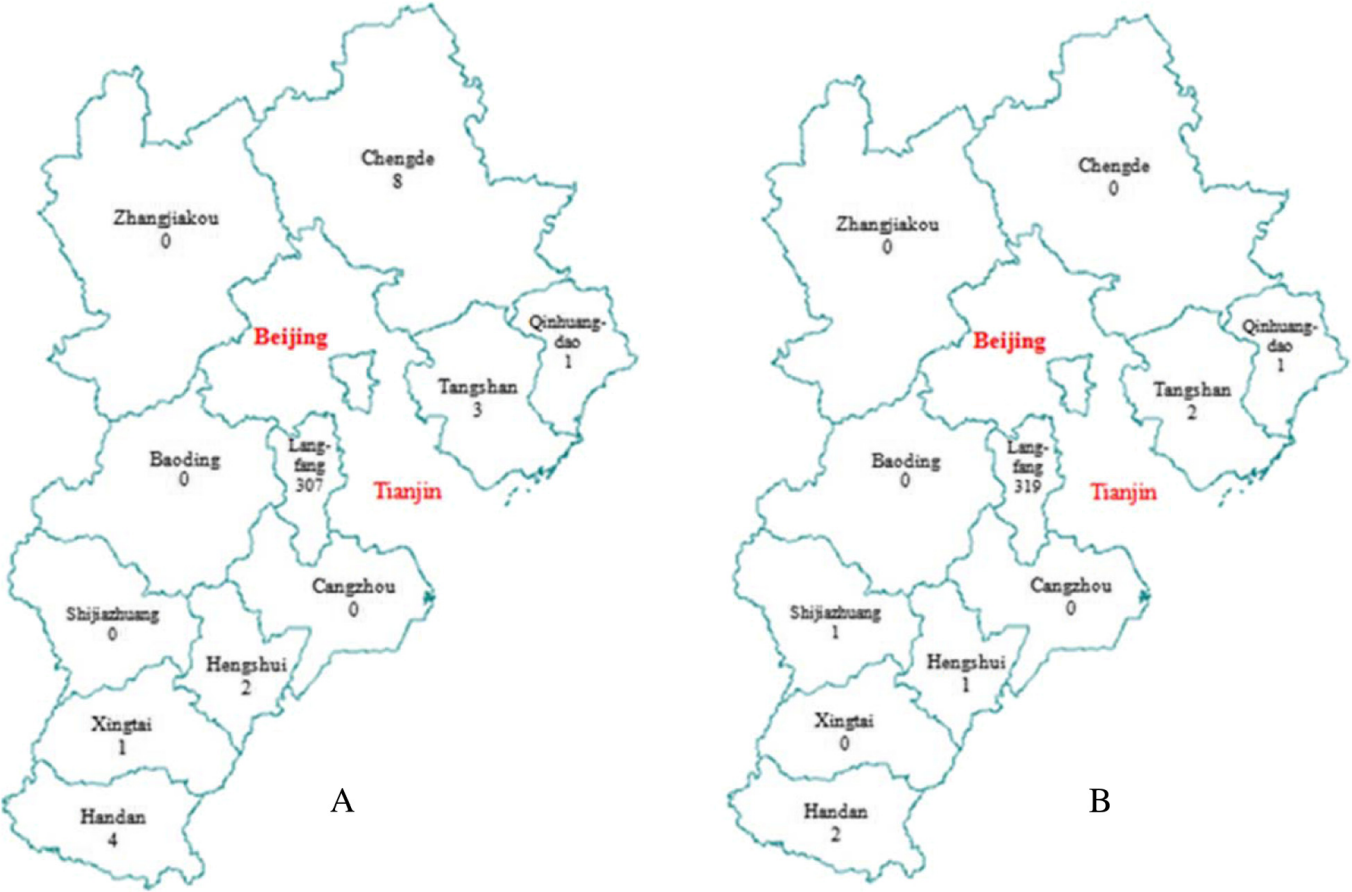
Area distribution of plasma-related HIV/AIDS cases in Hebei Province. Map **a** is a distribution of the residence locations of cases, and map **b** is that of plasma donation sites

During 1995–2013, there were 326 cases identified as HIV-positive arising from individuals who had donated blood at plasmapheresis centers in Hebei Province (Fig. 1). Among these, 94.2 % of individuals were residing in Langfang, 2.5 % in Chengde, 1.2 % in Handan, 0.9 % in Tangshan, 0.6 % in Hengshui, 0.3 % in Qinhuangdao, and 0.3 % resided in Xingtai. As to plasma donation site, 97.9 % of these individuals had donated blood in Langfang, 0.6 % in Handan, 0.6 % in Tangshan, 0.3 % in Hengshui, 0.3 % in Qinhuangdao, and 0.3 % had donated in Shijiazhuang. According to data obtained from tracking the source of infection, HIV infections first emerged in October 1994 [5]. All plasmapheresis centers were closed in April 1995; therefore, the transmission period was approximately 6 months. In the city of Langfang, the major transmission site, all plasmapheresis centers were closed in February 1995; therefore, the transmission period in Langfang was only 4 months.

### Annually reported cases

Among the 326 HIV/AIDS cases, the first case was identified on 26 January 1995, and the last case was identified on 28 February 2012. After the living subsidy policy was enacted in January 2012 for previously reported blood transmission cases, two plasma donor-related cases were diagnosed on 23 and 28 February 2012, in two individuals born in 1972 and 1971, respectively. Both of these cases were male, and their CD4 cell counts were 3 cells/mm^3^ and 85 cells/mm^3^, respectively, at diagnosis. Through 31 December 2013, no plasma donor-related HIV/AIDS cases had been diagnosed for a successive 22 months (651 days). The cases identified between 1995 and 1998 were all HIV-infected individuals (asymptomatic HIV carriers), and AIDS had begun to be identified beginning in 1999. As shown in Table 1, 146 cases from the general survey were identified in 1995, accounting for 44.8 % of the total. This data demonstrates that possibly 55.2 % of paid blood donors did not participate in the initial screening for various reasons.

**Table 1:** Identification, survival and death rate of plasma-related HIV/AIDS cases from 1995 to 2013

Year	Symptom when identified			Death & survival						HAART coverage	
	Number of HIV/AIDS cases reported	Number of HIV reported	Number of AIDS cases reported	Survival number of HIV/AIDS cases	Number of deaths	Death rate (%)	Accumulative death number	Accumulative death rate (%)	Accumulative survival rate (%)	Treated number	Coverage rate (%)
	(a)	(b)	(c)	(d)	(e)	(f)	(g)	(h)	(i)	(j)	(k)
1995	146	146	0	146	0	0.0	0	0.0	100.0	0	0.0
1996	1	1	0	147	0	0.0	0	0.0	100.0	0	0.0
1997	0	0	0	147	2	1.4	2	0.6	99.4	0	0.0
1998	2	2	0	147	2	1.4	4	1.2	98.8	0	0.0
1999	7	6	1	152	4	2.6	8	2.5	97.6	0	0.0
2000	16	14	2	164	9	5.5	17	5.2	94.8	0	0.0
2001	10	7	3	165	11	6.7	28	8.6	91.4	0	0.0
2002	10	7	3	164	18	11.0	46	14.1	85.9	0	0.0
2003	13	7	6	159	18	11.3	64	19.6	80.4	16	10.1
2004	33	19	14	174	24	13.8	88	24.5	75.5	47	27.0
2005	36	13	23	186	26	14.0	114	35.0	65.0	60	32.3
2006	14	7	7	174	8	4.6	122	37.4	62.6	63	36.2
2007	8	0	8	174	9	5.2	131	40.2	59.8	88	50.6
2008	9	1	8	174	13	7.5	144	44.17	55.8	97	55.8
2009	6	0	6	167	11	6.6	155	47.6	52.5	109	65.3
2010	7	2	5	163	6	4.7	161	49.4	50.6	115	70.6
2011	6	2	4	163	9	5.5	170	52.2	47.9	124	76.1
2012	2	0	2	156	4	2.6	174	53.4	46.6	127	81.4
2013	0	0	0	152	5	3.3	179	54.9	45.1	127	83.6

Notes: 1. (f) = (e)/(d)*100 %; 2. (h) = (g)/326*100 %; 3. (i) = 100 %-(h); 4. (k) = (j)/(d)*100 %

### Promptness of case identification

**Fig. 2:**
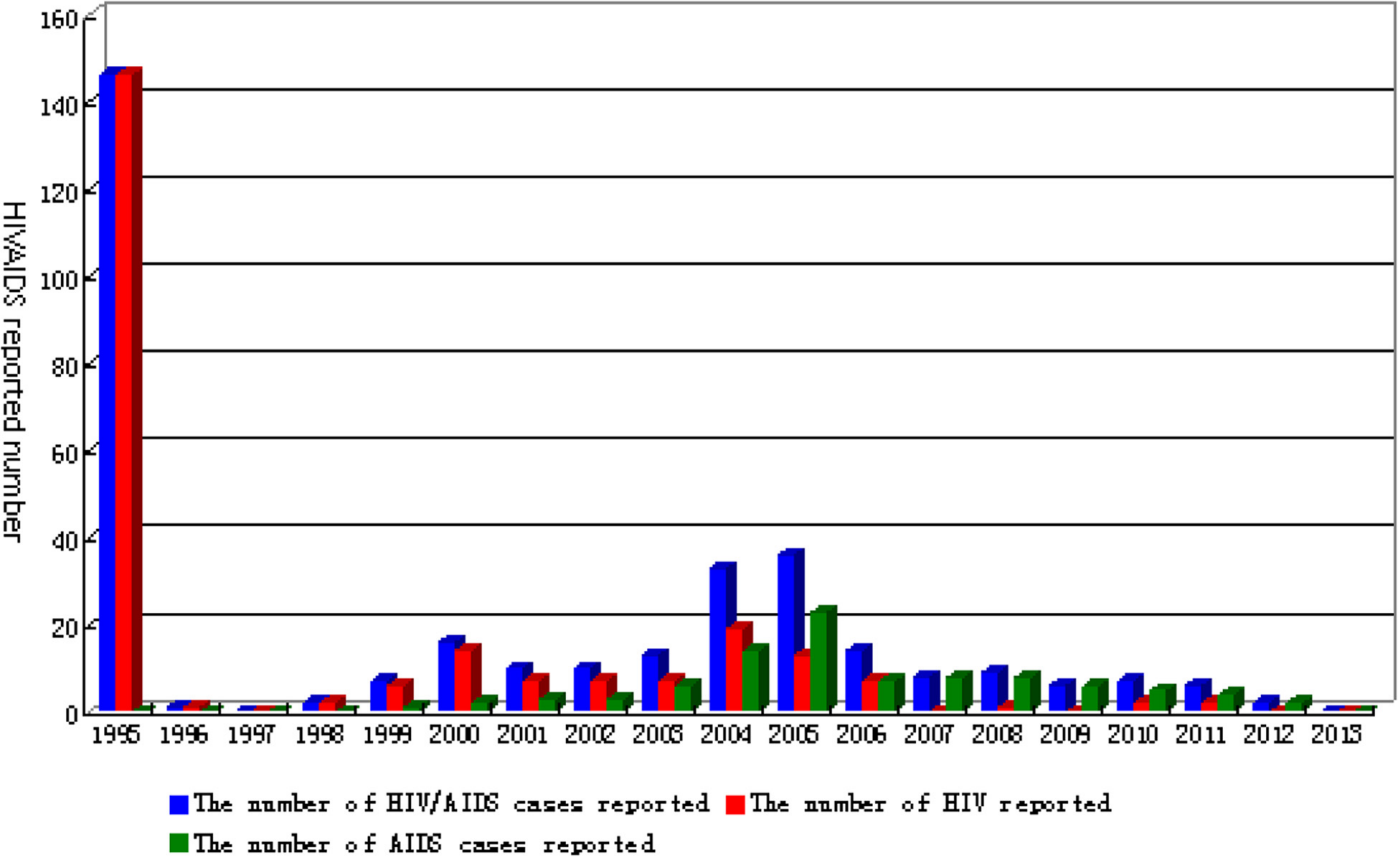
Distribution of plasma donor-related HIV/AIDS cases reported from 1995 to 2013 in Hebei Province

**Fig. 3:**
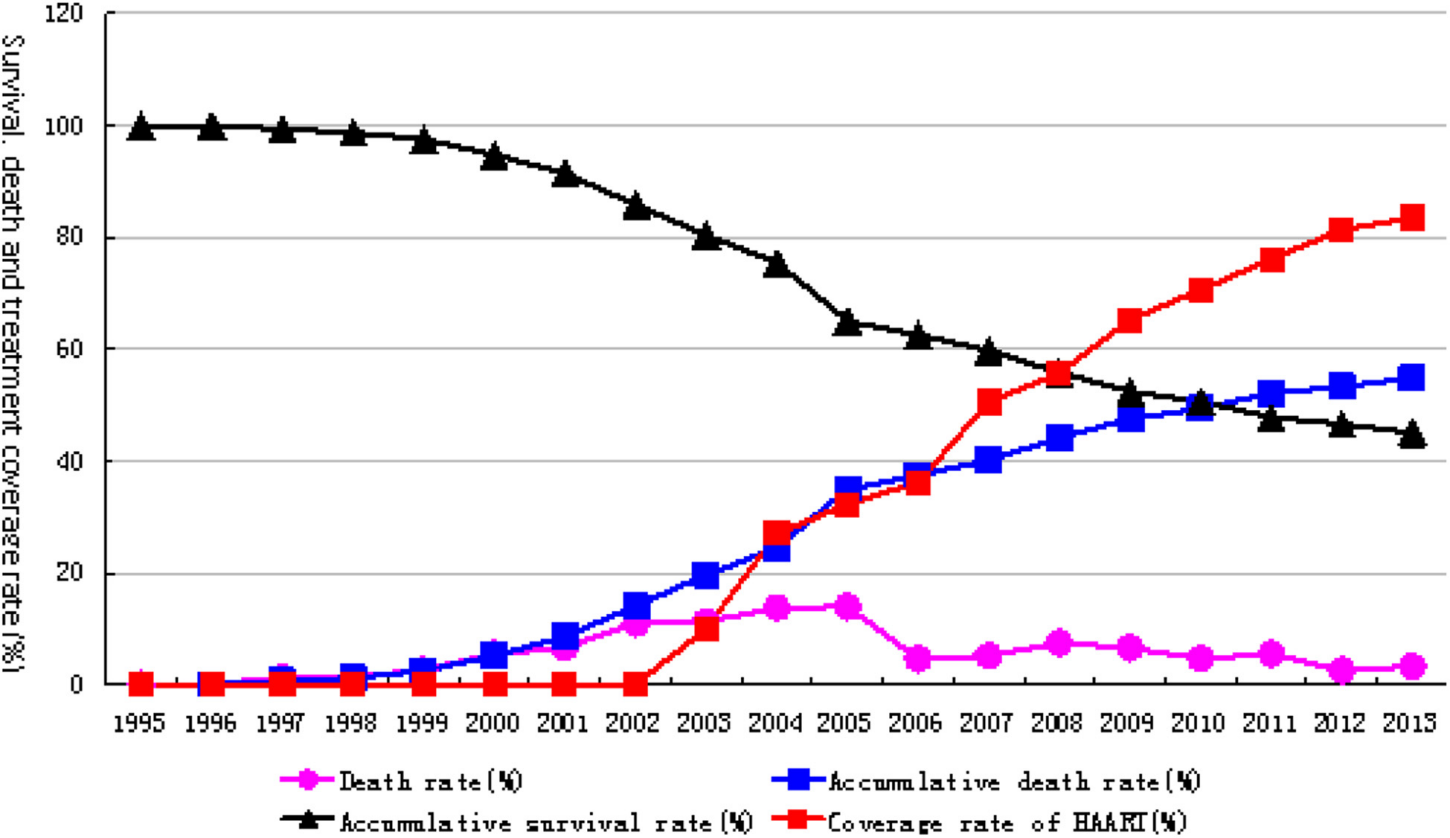
HAART coverage rate and changes in cumulative survival rate, cumulative mortality rate, mortality rate and antiviral therapy, from 1995 to 2013 in Hebei Province

Among the 326 HIV-positive cases, 234 cases were HIV-infected (asymptomatic HIV carriers) when identified, accounting for 71.8 %; 92 cases had AIDS when identified, accounting for 28.3 %. We deemed the HIV-positive cases identified in 1995 to be those cases identified in the same year as infection, with a total of 146 (44.8 %). The number of cases identified >1 year (after 1996) after infection was 180 (55.2 %), >5 years (after 2000) after infection was 170 (52.2 %), and >10 years (after 2005) after infection was 88 (27.0 %). Of those HIV cases identified in the same year as infection, 100 % (146/146) were asymptomatic HIV carriers. Of the HIV cases identified 1–4 years after infection, 90 % were asymptomatic HIV carriers (9/10). Of those cases identified 5–9 years after infection, 65.85 % were asymptomatic HIV carriers (54/82). Finally, of those cases identified 10–14 years after infection, 28.4 % were asymptomatic HIV carriers (25/88). These data are summarized in Table 1, and in Figs. 2 and 3.

### Survival rate

From all-cause mortality results measured in the year of infection, the data indicate that deaths began to occur in 1997 (mortality 1.4 %). Peak mortality took place from 2000 to 2005 (i.e., 6–10 years after infection). Up to 2013, the cumulative number of deaths was 179, a cumulative mortality rate of 54.9 %. Cumulative survival was 147 cases, a survival rate of 45.1 %. The mortality, cumulative mortality, and cumulative survival rate curves from 1995–2013 are shown in Table 1 and Fig. 3.

### Sexual transmission between couples

Among the 326 HIV/AIDS cases, 14 cases (3.1 %) were unmarried at the time they were informed of infection. Of the 312 HIV/AIDS cases who were married, the number of involved spouses or regular sexual partners was 314 (one infected individual had three sexual partners). Excluding 66 spouses who were not tested, 176 spouses had a history of exposure to a plasma donation. One spouse had sexual transmission outside of marriage, 71 spouses met the monitoring conditions for couple transmission, and 8 spouses tested positive for HIV antibody. Overall, the couple transmission rate was 11.3 %. Among the initial HIV/AIDS cases identified in 1995 (in the same year as infection), there were 30 cases of spouses who met the monitoring conditions for couple transmission. Until 2013, there was one case of couple transmission, and the transmission rate was 3.3 %. Among the HIV/AIDS cases identified from 1999 to 2012 (4–17 years after infection), there were 41 cases of spouses who met the monitoring conditions for couple transmission. Until 2013, there were seven cases of couple transmission and the transmission rate was 17.1 %, shown in Table 2.

**Table 2:** Couple transmission and mother-to-infant transmission in plasma donor-related HIV/AIDS cases

Year	Couple transmission detection		Mother-to-infant transmission detection	
	Number of spouses meeting couple transmission detection conditions	HIV-infected spouses until 2013	Number of children with possible mother-to-infant transmission	Mother-to-infant transmission number until 2013
1995	30	1	2	2
1996	0	0	0	0
1997	0	0	0	0
1998	0	0	0	0
1999	2	0	0	0
2000	5	3	0	0
2001	5	0	1	1
2002	0	0	2	1
2003	3	0	0	0
2004	7	1	0	0
2005	6	0	3	0
2006	5	1	4	1
2007	2	0	1	0
2008	0	0	0	0
2009	3	0	0	0
2010	1	0	0	0
2011	1	1	0	0
2012	1	1	0	0
2013	0	0	0	0
Total	71	8	13	5

### Mother-to-infant transmission

Among the 326 plasma donor-related HIV/AIDS cases, there were 171 female cases, 170 married cases, and 11 cases (6.6 %) that were not investigated after detection of HIV infection. Among another 159 female HIV/AIDS cases who were married and capable of bearing children, 145 individuals (91.2 %) did not have any children after being informed of HIV infection and 14 individuals (8.8 %) gave birth to a total of 19 children after being informed of infection. Blood samples were collected from 13 children of women infected with HIV and test, and five children tested positive for HIV antibody. The mother-to-infant transmission rate was 38.5 %, as shown in Table 2. The mothers of the five HIV-positive children were themselves identified as infected with HIV on 26 February and 6 March 1995, 2 March 2001, 1 April 2002, and 7 June 2006. The five children were born on 10 February 1995, 1 August 2007, 7 March 2001, 22 March 1996 and 18 May 2003, and were identified to be HIV-positive on 4 March 2004, 8 April 2009, 5 July 2003, 2 April 2005 and 2 April 2005, respectively. All mother-to-infant transmission cases appeared prior to the start of mother-to-infant HIV transmission prevention policies adopted in local regions.

To trace the possibility of HIV-positive males transmitting HIV to their spouses and subsequently to their children via mother-to-infant transmission, we carried out an analysis of the 155 male plasma donor-related cases. We investigated possible transmission factors for the 144 spouses of the 142 married cases therein and found that 76 cases had histories of plasma donation, ten cases were not investigated, one case had engaged in extramarital sexual behavior, one case had no possible transmission route and another 56 female spouses in which couple transmission was the sole transmission factor. Among these 56 female spouses, 42 consented to HIV testing and five were found to be HIV-positive. Four female spouses did not bear any children after being informed of infection with HIV, and one did not consent to HIV testing. Third-generation transmission to infected children via mother-to-infant transmission was not detected.

### Antiviral therapy

With respect to HAART measures begun in 2003, coverage was relatively low owing to an insufficient supply of antiviral drugs in the early stages of the government program. With the increased number of patients, improved antiviral drug supply, and modified therapeutic strategy, the HAART coverage rate has increased over time and the death rate has declined. Approximately 45.1 % of HIV/AIDS patients have survived 18 years after infection with HIV, as shown in Table 1 and Fig. 3.

### HIV and subtypes

**Fig. 4:**
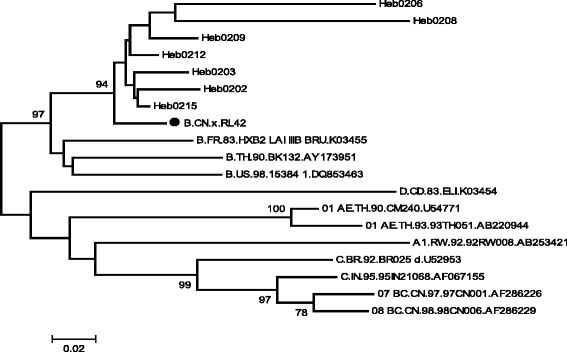
Phylogenetic analysis of HIV-1 *env* for international reference strains and Hebei strains from former paid blood donors ● indicates Thai subtype B of international HIV-1 reference

All 326 plasma donor-related HIV/AIDS cases and second-generation cases were infected with HIV-1. PCR amplification was conducted for blood samples from seven cases; the PCR products were then sequenced and used to create a phylogenetic tree, seen in Fig. 4. The prevalent HIV-1 strain belongs to B’ subtype, particularly in combination with Yunnan Ruili strain RL42 *env* (Thai subtype B).

## Discussion

In early 1983, Gury reported HIV infections among paid blood donors [6], but the report did not draw attention to infection among paid blood donors at a global level. As a result, the prevalence of HIV infection among paid blood donors continued to increase globally. In 1985, Wendel et al. reported HIV infections among paid blood donors in Brazil [7]. Until 1995, similar infections had been reported in Greece, Western Germany, California in the United States, Mexico, India, Spain, Thailand, Cambodia, Zaire and Nigeria [8–10]. In 1985 and 1993, hepatitis C virus and malaria were found to be prevalent among plasma donors in Hebei Province in China [1–3], and the clear conclusion was drawn that the cause was cross-infection during blood collection. However, the prevalence of HIV in this population was not established until 1995.

In 1995, when HIV was discovered to be prevalent among plasma donors in Hebei, all plasmapheresis centers in the province were closed that same year. Subsequently, the prevalence of HIV acquired from cross-infection among blood donors ceased immediately. However, owing to the long incubation period of AIDS, social discrimination, and panic related to the disease, several donors concealed their histories as former paid blood donors so they were not included in the initial HIV screening conducted in 1995. Therefore, from 1996 to 2012, plasma donor-related HIV/AIDS cases were still being identified. In particular, between 2000 and 2006, HIV-infected donors entered the symptomatic period of disease. Because clinicians had been widely trained and the HIV monitoring network had gradually improved, in combination with collective screening activity from December 2004 to February 2005, the identification of HIV/AIDS cases peaked from 2000 to 2006. After 18 years of monitoring, 326 HIV/AIDS cases have been found to be related to blood plasma donation at plasmapheresis centers in Hebei Province. Additionally, 8 cases originated from couple transmission and five cases from mother-to-infant transmission. Second-generation disease transmission accounted for 4.0 % of cases. The total number of first- and second-generation cases is 339. All first- and second-generation plasma donor-related HIV/AIDS cases were infected with HIV-1.

The overall couple transmission rate in plasma donor-related HIV/AIDS cases was 11.3 %. In 1995, in the same year as infection, the couple transmission rate was 3.3 %. The couple transmission rate in cases identified 4–17 years after infection was 17.1 %. The couple transmission rate of the former was approximately 80.5 % lower than that of the latter. Couple transmission rates in cases identified 4–17 years after infection were consistent with the natural couple transmission rate of 17.3 % in situations of ignorance about HIV status [11]. This demonstrates that being informed earlier about the HIV-positive status of the spouse or sexual partner is favorable for the HIV-negative partner to change their sexual behavior, thereby reducing the risk of HIV transmission. Therefore, it is clear that early identification and early notification with respect to HIV infection plays an important role in controlling HIV transmission. Of single cases, 3.1 % remained unmarried after being informed of HIV infection, which also effectively prevented the occurrence of couple transmission.

This study also revealed that 91.2 % of married female plasma donor-related HIV cases did not bear children after being informed of infection, which largely decreased the chances of mother-to-infant transmission. Therefore, diagnosis in the early stages of infection may reduce the occurrence of mother-to-infant transmission. In situations where the mother was ignorant about her HIV-positive status and did have children, the mother-to-infant transmission rate was 38.5 %. According to Chen et al., in situations where mother-to-infant transmission prevention measures were not applied, the mother-to-infant transmission rate of mothers infected by blood transfusion was 32.1 % [12]. According to Scarlatti et al., approximately 11.0 %–60.0 % of HIV-positive pregnant women demonstrate mother-to-infant transmission. This rate has been reported as 20.0 %–30.0 % in the United States, 15.0 % in Europe, and 50.0 % in Africa [13]. According to Li Guanhan [14], the mother-to-infant transmission rate of HIV-1 is 35.0 % in ten provinces in China. This result is nearly identical to a report suggesting that the rate is 41.7 % in Henan Province, 33.3 % in Yunnan Province, 27.3 % in Xinjiang Province [14].

Until 2013, the cumulative mortality rate and survival rate of plasma donor-related HIV/AIDS cases was 54.9 % and 45.1 %, respectively. Owing to ongoing improvements in HAART coverage, the survival period for HIV-positive individuals has been extended. Before HAART measures were instituted, the average survival time for former blood donors infected with HIV was 9.58 years [15], and that of HIV-infected blood transfusion recipients was 9.92 years [16]. Once the rate of HAART coverage had reached 70.6 % of all HIV cases, 50.6 % of these cases had survived until 15 years after infection. When the HAART coverage rate reached 83.6 % of all HIV cases, 45.1 % of these cases survived until 18 years after infection. According one study in Uganda, it was found that if the patient’s CD4 levels exceeded 150 cells/mm^3^, HAART treatment would extend life expectancy to nearly that of a normal healthy individual [17]. Thus, it is clear that early therapy and enhancing the HAART coverage rate can greatly decrease the mortality rate and extend the life span of HIV-infected individuals.

## Conclusions

In this accident of HIV-infection among plasma donors in Hebei Province, a total of 339 direct and second-generation cases have been identified over 18 years of monitoring. Favorable clinical results have been achieved using intervention measurements and antiviral therapy.
